# Relationship between Psychological Distress and Prolonged Sedentary Bouts in the Elderly: Four Period Analysis

**DOI:** 10.3390/healthcare9060676

**Published:** 2021-06-04

**Authors:** Yutaka Owari

**Affiliations:** Shikoku Medical College, Utadu 769-0205, Kagawa, Japan; seikotsuin@nifty.com; Tel.: +81-87-41-2320

**Keywords:** psychological distress, prolonged sedentary bouts, the elderly, structural equation modelling (SEM)

## Abstract

Background: Too much sitting is associated with low mental health in elderly individuals. We clarified the relationship between psychological distress and the rate of prolonged sedentary bouts (PSBs) among the elderly over four periods. Methods: In a secondary analysis, a sample population of 68 adults aged 65 years or older in Japan was used. The following proxy variables were used: PSB (mental health) and the Kessler 6 scale; K6 scores (psychological distress). Results: Using the cross-lagged effects models, from “2016 K6” to “2017 PSB” (*p* = 0.004), from “2017 K6” to “2018 PSB” (*p* < 0.001), and from “2018 K6” to “2019 PSB” (*p* = 0.021) were all significant; however, the reverse were not all significant in one period. In four periods, from “2016 PSB” to “2019 K6” (*p* = 0.025) was significant; however, the reverse was not significant. Fit indices were obtained: χ^2^ = 7.641 (*p* = 0.182), goodness of fit index (GFI) = 0.891, comparative fit index (CFI) = 0.901, and root mean square error of approximation (RMSEA) = 0.121 in structural equation modelling. Conclusions: Psychological distress may affect the rate of PSB after one year, and the rate of PSB may affect the rate of psychological distress after three years in elderly individuals.

## 1. Introduction

Physical inactivity and sedentary behavior (SB) are major risk factors to chronic illness and obesity, which cause premature death and are socially and economically burdensome. Regular physical activity (PA) is now clearly recognized for its protective effect against non-communicable diseases, which are associated with lower mortality and improved quality of life and are therapeutically effective for many chronic diseases. The proper monitoring of PA and SB is clearly a public health concern.

Excessive sitting is associated with low mental health in the elderly population and has been shown to be associated with decreased cognitive function [1], the risk of developing dementia [2], and reduced health-related quality of life [3]. Japan has one of the highest aging populations in the world, and, according to the Cabinet Office of the Government of Japan, the population aged 65 or older is 28.1% [4]. Therefore, maintaining the health of the elderly is important and applies to other countries with a rapidly growing aging population.

A prolonged sitting position has been shown to increase the risk of diabetes, metabolic syndrome, heart disease, and cancer [5,6,7]. Although studies have shown that shorter rest periods increase HDL cholesterol levels [8], reduce pain and improve mental health [9], other studies have shown the opposite results [10,11]. However, the cause is unknown. In addition, recent studies have shown that even with similar total sitting times, health risks vary depending on whether sedentary behavior is disrupted [12,13]. Those with fewer interruptions in sedentary behavior often have more biomarkers for cardiovascular metabolic disease than those with frequent disruptions [14].

Therefore, we used the cross-lagged effect model. This model, which uses longitudinal data at two or more time points obtained by the panel survey, is effective for examining “the causal relationship”. If the variable X (for example, prolonged sedentary bouts: PSBs) at the first time point is X_1_, the variable Y (for example, psychological distress: PD) is Y_1_, the variable X at the second time point X_2_, the variable Y is Y_2_. The variable at the second time point is predicted by the variable at the first time point, but X_1_ is statistically controlled in X_2_, Y_1_ is statistically controlled in Y_2_, and then the lagged effect of Y_1_ on X_2_ and that of X_1_ on Y_2_. The lagged effect will be considered. Additionally, by assuming the correlation between the error variables, the confounding of the third variable can be assumed. The idea of examining causality from the cross-lagged effect of controlling prior values corresponds to “Granger causality” [15], and since the possibility of the influence of residual variables cannot be ruled out, “in sense of Granger”, it creates a causal relationship with the word. However, this analysis is effective in examining the direction of causal relationships between variables, and can strongly show that, for example, X affects Y and not the other way around.

Here, we clarified the relationship between PD and the rate of PSBs over four periods. The following was hypothesized: in the one period, an improvement in PD causes a decrease in PSBs, but the reverse occurs in the four periods.

## 2. Materials and Methods

### 2.1. Study Design

Using a secondary analysis from a previous report [16], a longitudinal study was performed using “four period” panel data. The following proxy variables were used: PSBs (based on 1.5 MET sessions or more lasting 30 min or more) and PD (based on Kessler 6 scale (K6) scores). The survey method was evaluated by using a 3-axis accelerometer and a self-managed questionnaire.

Our study was approved by the Ethics Review Board of Shikoku Medical College (approval numbers: H27-3, H27-7, H28-6, R01-3-001), and written informed consent was obtained from each subject.

### 2.2. Participants

**Figure 1:**
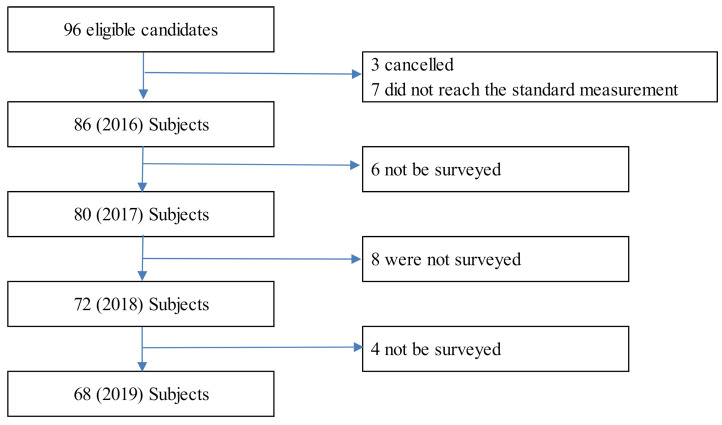
Follow-up chart.

The flow chart of the participants is shown in Figure 1. Appropriate sample sizes have not yet been established for the cross-lagged and synchronous effects models. Since the number of samples was 68, we used a simple model and resampled by 1000 parametric bootstraps.

The first phase of the study involved a survey that assessed 96 healthy elderly individuals participating in a college health class in Utadu, Japan (population, 18,500) between 20 July and 10 September 2016. Three withdrew from the study and seven were excluded from the analysis since they were not in compliance with standard physical activity measurements. Therefore, we used the data from the remaining 86 respondents. In the second phase, similar monitoring was carried out between 20 July and 15 September 2017. Six of these respondents were excluded from the survey due to incomplete data. Consequently, information based on 80 participants was used. Similar monitoring was carried out between 29 April and 31 May 2018 in the third stage. Eight of these respondents could not be surveyed. Therefore, data based on 72 participants were used. In the fourth phase, a similar follow-up was conducted between 30 July and 19 September 2019. Eight of these respondents were unable to obtain survey results. Therefore, data based on 68 participants (average age, 73.6 ± 5.5 years old; K6 score, 2.29 ± 2.03) were used.

### 2.3. Clinical Parameters and Measurement

The following anthropometry and body composition parameters were evaluated between 2016 and 2019 [16]: age (years), height (cm), body weight (kg), and body mass index (BMI): kg/m^2^).

### 2.4. Psychological Distress

The K6 score data have been cited in previous studies. PD was assessed by using six items from the Japanese version of the K6 scale. K6 is a self-administered questionnaire developed by Kessler as a screening test for PD and is also able to identify factors related to PD, which may provide insight on how to improve mental health. Six points were answered on the Likert scale, and each item’s answer was converted to a score of 0–4 points. The questionnaire consists of 6 questions: How often did you feel the following last month? (1) Nervous, (2) Desperate, (3) Restless or fidgety, (4) Depressed to the point where nothing can cheer you up, (5) Everything was an effort, (6) worthless. The subjects were asked to answer by choosing from “always” (4 points), “mostly” (3 points), “sometimes” (2 points), and “a little of the time” (1 point) and “no time” (0 points). We evaluated the PD level as a total score. Therefore, the score ranged between 0 and 24. Data from a 2019 survey were also added to this study.

### 2.5. Physical Activity

Physical activity data were cited in a previous paper. Data were recorded by using a 3-axis accelerometer (Active Style Pro HJA-750C, Omron Healthcare, Tokyo, Japan) for 7 consecutive days. Subjects were requested to wear this device at all times except during certain activities, such as swimming or bathing. The standard deviation of the 10-s data was defined as the mean of the acceleration. If there was a lack of data, data obtained on Saturday or Sunday were also used to satisfy the requirement of wearing the device for more than 10 h per day. The rate of PSBs was defined as follows: (the periods during which sedentary behavior lasts 30 min or more a day)/(waking time in a day, minutes) × 100.

### 2.6. Statistical Analyses

First, the Levene test and Brown–Forsythe test were performed to test the uniformity of population variance between the data of the four groups (2016, 2017, 2018, 2018). As a result, the uniformity of population dispersion among the four groups was proved.

Second, we used structural equation modeling (SEM) to clarify the “causal relationship” between PD and PSBs. First, to work out whether the variables from the primary survey were sufficiently predictable within the second, third, and fourth surveys, PD and PSB variables in 2016 were compared with those in 2017, 2018 and 2019. Second, to evaluate the “causal relationship” between PD and PSBs, we used the cross-lagged effects model. Third, we compared PD and PSBs in 2017 with those in 2018. Fourth, PD and PSBs in 2018 were compared with those in 2019. Fifth, we compared PD and PSBs in 2016 with those in 2019. Finally, to test the fit of those models, the subsequent indices were used: χ^2^ (the model conformed to the info if *p* > 0.05), goodness of fit index (GFI, 0 to 1; corresponds to determination coefficient in multivariate analysis with acceptable fit at values 0.95 or greater), comparative fit index (CFI, 0 to 1; an indicator that corrects the effect of the amount of knowledge with acceptable fit at values at 0.95 or greater), and root mean square error of approximation (RMSEA, an index that shows the deviation between model distribution and true distribution as an amount per one degree of freedom with acceptable fit at values but 0.05 [17]). We performed all calculations using SPSS version 26 and AMOS version 26 (IBM, Chicago, IL, USA).

## 3. Results

Table 1 presents the clinical profiles of the enrolled participants.

We performed a stepwise regression analysis using K6 scores and PSBs as the purpose variable, and age, sex, and BMI as explanatory variables to adjust for confounding factors.

**Figure 2:**
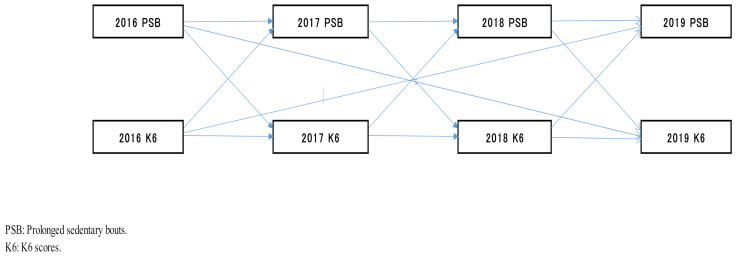
Cross-lagged effects model.

First, the K6 scores and the PSBs in 2016 were correlated with the K6 scores and the PSBs in 2017, respectively (PD, standardization factor (β) = 0.831; PSB, β = 1.010; *p* < 0.001 for both), the K6 scores and the PSBs in 2017 were correlated with the K6 scores and the PSBs in 2018, respectively (PD, β = 0.909; PSB, β = 0.818; *p* < 0.001 for both), and the K6 scores and the PSBs in 2018 were correlated with the K6 scores and the PSBs in 2019, respectively (PD, β = 0.564; PSB, β = 0.812; *p* < 0.001 for both) (Figure 2, Table 2).

Second, the path of the model from the K6 scores in 2016 to the PSBs in 2017 was significant (β, 0.165; *p* = 0.004); in contrast, the reverse was not (β, 0.100; *p* = 0.141). Therefore, the K6 scores in 2016 may exert a causal effect (0.165) on the PSBs in 2017 using the cross-lagged effects model (Figure 2, Table 2).

Third, the path of the model from the K6 scores in 2017 to the PSBs in 2018 was significant (β, 0.238; *p* < 0.001); though the reverse was not (β, 0.040; *p* = 0.512). Hence, the K6 scores in 2017 may exert a causal effect (0.238) on the PSBs in 2018 by the cross-lagged effects model (Figure 2, Table 2).

Fourth, in the cross-lagged effects model, the path of the model from the K6 scores in 2018 to the PSBs in 2019 was significant (β, 0.271; *p* = 0.021); although, the reverse was not (β, 0.224; *p* = 0.055). Thus, the K6 scores in 2018 may exert a causal effect (0.271) on the PSBs in 2019 (Figure 2, Table 2).

**Figure 3:**
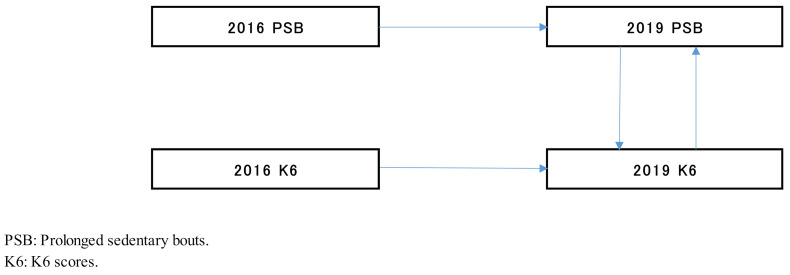
Synchronous effects model.

Fifth, in the cross-lagged effects model, the path of the model from the K6 scores in 2016 to the PSBs in 2019 was not significant (β, 0.218; *p* = 0.073); however, the reverse was significant (β, 0.260; *p* = 0.025) (Figure 2, Table 2). Using the synchronous effects model, the paths from the K6 scores to the PSBs and the PSBs to the K6 scores were significant (β, 0.328, *p* = 0.018; β, 0.598, *p* < 0.001) in 2019 (Figure 3, Table 2). The PSBs to the K6 has a stronger influence on the result than the K6 to the PSBs since the following was observed: from the PSBs to K6 (β: 0.598) > from the K6 to the PSBs (β: 0.328). Therefore, the PSBs in 2016 may exert a causal effect (0.218) on the K6 scores in 2019.

Finally, to measure the fit of these models (excludes insignificant paths), the following fitness indexes were found: χ^2^ = 7.641 (*p* = 0.182), GFI = 0.891, CFI = 0.901 and RMSEA = 0.121.

## 4. Discussion

There were six main findings in our study. First, the 2016 PD was significantly correlated with the 2017, 2018, and 2019 PDs. Similarly, PSBs in the 2016 were significantly correlated with PSBs in the 2017, 2018 and 2019. These results showed that aging was not an important factor in our study. Although not performed in our study, we had to use a more accurate analysis, the “latent change model or latent curve model” [18].

Second, the 2016, 2017 and 2018 K6 scores may have affected the 2017, 2018 and 2019 PSBs [19]. Thus, improvements in PD may reduce the rate of PSBs after one year. This is consistent with the following report. One factor associated with motor motivation is a positive affect or emotion. Specifically, it is interest, contentment, and love [20]. Those with a high positive affect or emotion can be said to have good mental health. It is associated with health-promoting behavior. For example, positive emotions are associated with high levels of exercise (i.e., low sitting time) [21].

Fifth, analysis using the cross-lagged effects model indicated that the path from K6 in 2016 to PSBs in 2019 was not significant, despite influence from PSBs in 2016. In contrast, the path from the 2016 PSBs to the 2019 K6 score was significant. These results tell us that the 2016 PSBs may have influenced the 2019 K6 score. Therefore, using the cross-lagged and synchronous effects models, improving the rate of PSBs can reduce K6 after 3 years. These results are agreement with other studies which showed that high levels of physical activity reduce the likelihood of depression [22,23,24]. Understanding the physiological and biological mechanisms that lead to four-period sitting behavior is important but not yet sufficient. Prolonged sitting behavior may cause metabolic disorders such as the uptake of free fatty acids into muscle cells and the suppression of lipoprotein lipase activity required for HDL production [25]. In the sitting position, there is little contraction of the leg muscles and cardiovascular metabolism can be inactive [26]. A longitudinal study of residents reported that people with less physical activity (i.e., more sitting time) were more likely to be depressed than those with more [23,27]. This indicates a causal relationship that having more physical activity (i.e., less sitting time) means one is less likely to be depressed. Additionally, from the field of exercise physiology, the endorphin hypothesis, serotonin hypothesis, norepinephrine hypothesis, body temperature hypothesis, etc., have been reported as mechanisms by which exercise improves the symptoms of depression. Additionally, they may improve anxiety [28]. However, the mechanism by which psychological distress reduces sedentary behavior has not yet been fully elucidated.

Finally, a structural equation model was required to ensure that the measured model did not initially introduce any non-logical values and that it fitted well before interpreting the results. We obtained χ^2^ (*p* = 0.182 (>0.05)) in which the null hypothesis was that the constructed model was correct. Since the χ^2^ value was larger than a certain significance level, the model was not rejected. Therefore, it was adopted for this study. However, the model did not have a high fit (GFI, CFI, and RMSEA).

This study has some limitations. First, there may be a problem with “regression to the mean value”. That is, when carrying out longitudinal research with two periods, the high score of the initial research tends to be higher than the true score of the researched subject, and the low score of the research tends to be lower than the true score of the researched subject. The high score in the first research decreases in the second research; however, the low score in the first research increases. To mitigate this problem, the number of time points should be increased since the observed score fluctuates randomly about the true score. Thus, if the change is measured over a period of 3 time points or more, the problem due to regression to the mean value may be reduced.

Second, the study did not consider the intervention of a third variable. If a causal relationship between x and y is suspected, it can still be affected by an unknown third variable, such as socio-economic variables. Therefore, there was a significant difference between the K6 scores and PSBs; however, the model was not a good fit (GFI, CFI, and RMSEA). For example, we can consider variables such as physical health and socioeconomic status. In our previous study [29], the K6 scores were based on age, BMI (kg/m^2^), working hours (h/day), walking hours (min/day), sleeping hours (h/day), marital status (with or without a spouse), smoking status (smoker or non-smoker), and drinking status (drinker or non-drinker). Future studies should incorporate a third variable, z, into the model that may affect the causal estimation between x and y.

Third, as described below, many changes in physical activity due to COVID-19 have been reported, but we have not been able to fully examine this. A web survey of 1047 adults aged 18 and over worldwide from 6–11 April 2020 showed that the average weekly MVPA time was 36 per week compared to before the COVID-19 epidemic. Minutes (33.5%) decreased and sitting time increased by 3.1 h (28.6%) per day (IPAQ: International Physical Activity Questionnaire) [30]. Additionally, from a web survey of 1425 elderly Japanese people, the median MVPA time per week (IPAQ: International Physical Activity Questionnaire) was 240 min (90-90-) in January 2020 before the COVID-19 epidemic (480 min), April, while under the state of emergency was 180 min (60–315 min), and June, after the state of emergency was 270 min (100–550 min) [31].

Finally, because the sample size was less than 100, we used the maximum likelihood method, the bootstrap method, and a simple model. However, we need more samples in the future to use more complex models.

## 5. Conclusions

In the short term, improved PD may cause a decrease in PSBs, but in the long term, a decrease in PSBs may cause an improvement in PD. Our data suggest that PD may impact the rate of PSBs after one year, and PSBs may impact the rate of PD after three years. For the elderly, mental health may impact sitting behavior in the one period; however, in four periods, sitting behavior may impact mental health. Reduced sitting behavior may improve the mental health in the elderly. For future studies, the repercussions of sitting behavior on mental health and other factors that may improve the mental health of the elderly will be studied.

## Figures and Tables

**Table 1:** Clinical characteristics of enrolled subjects.

	2016			2017			2018			2019		
	Mean ± SD	Min.	Max.	Mean ± SD	Min.	Max.	Mean ± SD	Min.	Max.	Mean ± SD	Min.	Max.
Number of subjects	68 (men, 22)											
Age (years)	73.6 ± 5.5	68	88									
Height (cm)	157.4 ± 9.2	138.3	178.4	157.3 ± 9.0	138.1	178.2	157.1 ± 8.9	138.1	178.1	157.0 ± 8.8	138.0	178.0
BMI (kg/m^2^)	23.1 ± 2.5	13.9	29.1	23.4 ± 2.7	14.9	29.2	22.8 ± 2.8	15.2	30.1	22.7 ± 3.0	14.5	30.2
≤1.5 Mets (%/day)	55.5 ± 9.8	35.4	79.9	56.3 ± 11.1	20.9	75.4	55.4 ± 10.8	20.2	75.2	54.3 ± 10.7	20.2	75.2
PSB: Prolonged sedentary bouts (%)	14.7 ± 8.3	0.0	40.8	15.1 ± 8.3	1.9	41.6	14.6 ± 7.7	0.0	39.8	13.1 ± 7.9	0.0	36.5
K6 scores	2.52 ± 3.16	0	14	2.59 ± 3.23	0	14	2.32 ± 2.31	0	11	2.29 ± 2.03	0	10

PSB: Prolonged sedentary bouts; K6 scores: Kessler 6 scores; Min.: Minimum; Max.: Maximum.

**Table 2:** Maximum likelihood parameter estimates.

		Path	Standardization Factor (β)	*p*
aging		2016PSB → 2017PSB	1.010	**<0.001**
		2017PSB → 2018PSB	0.818	**<0.001**
		2018PSB → 2019PSB	0.812	**<0.001**
		2016K6 → 2017K6	0.831	**<0.001**
		2017K6 → 2018K6	0.909	**<0.001**
		2018K6 → 2019K6	0.564	**<0.001**
cross-lagged effects model	short-term	2016PSB → 2017K6	0.100	0.141
		2016K6 → 2017PSB	0.165	**0.004**
		2017PSB → 2018K6	0.040	0.512
		2017K6 → 2018PSB	0.238	**<0.001**
		2018PSB → 2019K6	0.260	0.055
		2018K6 → 2019PSB	0.218	**0.021**
	long-term	2016PSB → 2019K6	0.224	**0.025**
		2016K6 → 2019PSB	0.271	0.073
synchronous model		2019PSB → 2019K6	0.598	**0.018**
		2019K6 → 2019PSB	0.328	**0.032**

*p*: Bold is significant.

## Data Availability

Due to the nature of this research, participants of this study did not agree for their data to be shared publicly, so supporting data is not available.
